# Spill the load: Mixed evidence for a foveal load effect, reliable evidence for a spillover effect in eye-movement control during reading

**DOI:** 10.3758/s13414-019-01689-5

**Published:** 2019-03-04

**Authors:** Eva Findelsberger, Florian Hutzler, Stefan Hawelka

**Affiliations:** Centre for Cognitive Neuroscience, University of Salzburg, Hellbrunnerstr. 34, 5020 Salzburg, Austria

**Keywords:** Reading, Eye movements, Word frequency

## Abstract

It has been hypothesized that the processing difficulty of the fixated word (i.e., “foveal load”) modulates the amount of parafoveal preprocessing of the next word. Evidence for the hypothesis has been provided by the application of parafoveal masks within the boundary paradigm. Other studies that applied alternative means of manipulating the parafoveal preview (i.e., visual degradation) could not replicate the effect of foveal load. The present study examined the effect of foveal load by directly comparing the application of parafoveal masks (Exp. 1) with the alternative manipulation of visually degrading the parafoveal preview (Exp. 2) in adult readers. Contrary to expectation, we did not find the foveal-load interaction in the first experiment with traditional letter masks. We did, however, find the expected interaction in the second experiment with visually degraded previews. Both experiments revealed a spillover effect indicating that the processing of a word is not (always) fully completed when the reader already fixates the next word (i.e., processing “spills over” to the next word). The implications for models of eye-movement control in reading are discussed.

## Introduction

The preprocessing of words that are not yet fixated (i.e., parafoveal preprocessing) speeds up word recognition, and hence serves fast and fluent reading (Rayner, 1998, 2009; Schotter, Angele, & Rayner, 2012). The amount of the speedup due to parafoveal preprocessing has been dubbed the *preview benefit*. Several factors may influence the magnitude of the preview benefit. One such factor could be the ease of recognizing the currently fixated word: the easier it is to recognize the currently fixated word, the more resources are available for preprocessing the upcoming word. This assumption has been termed the *foveal load hypothesis*.

A seminal study on the foveal load hypothesis by Henderson and Ferreira (1990) addressed the question of whether a high foveal load (i.e., a difficult foveal word) decreases the preprocessing of the parafoveal word and – as a result – diminishes the preview benefit. The main experimental manipulation of the study revolved around the difficulty of the pretarget word (word_n-1_), which preceded the target word (word_n_). The manipulation was varying word frequency. The rationale was that a high-frequency word induces a low foveal load; a low-frequency word induces a high load. The dependent measure was the magnitude of the preview benefit on the target word (i.e., word_n_).

The magnitude of the preview benefit was estimated by the application of the *boundary paradigm* (Rayner, 1975). This paradigm makes it possible to experimentally manipulate the characteristics of the parafoveal preview. To illustrate, in a sentence an invisible boundary is placed prior to a theoretically relevant target word. As long as the reader fixates to the left of the boundary, an experimentally manipulated parafoveal preview is provided. Commonly, a string of letters – (partially) different from the letters of the target word – is used to cover the target word (henceforth referred to as *parafoveal mask*). When the reader’s eyes cross the boundary, the mask is replaced by the target word (the change itself is invisible for the reader due to saccadic suppression – Matin, 1974 – but a proportion of readers is usually aware that fixated words do not match the parafoveal previews). Parafoveal masking can be used to estimate the magnitude of the preview benefit by subtracting the reading times of targets words after a valid preview from the reading times of the target words whose preview was (partially) masked. Specifically, Henderson and Ferreira (1990) presented visually similar previews (i.e., the preview is partially valid insofar as the first three letters were the same as in the actual target word; the remaining letters were replaced with visually similar letters, e.g., target word: *despite*, preview: *desqlda*) and visually dissimilar previews (invalid preview, e.g., target: *despite*, preview: *zqdloyv*).

Henderson and Ferreira (1990) reported effects compatible with the *foveal load hypothesis*. If the foveal load induced by the pretarget was low (i.e., word_n-1_ was high frequent), then a preview benefit was observed, that is, the mean processing time of the target word was shorter after a valid (and a partially valid) preview than after an invalid (i.e., the dissimilar) preview. No preview benefit was observed if the foveal load induced by the pretarget was high (i.e., word_n-1_ was low frequent). Technically speaking, Henderson and Ferreira (1990) reported a LOAD by PREVIEW interaction. Such an interaction was reported for first fixation duration and gaze duration.

Recent evidence questioned the suitability of parafoveal masks for the estimation of the preview benefit (e.g., Hutzler, Schuster, Marx, & Hawelka, in press; Hutzler et al., 2013; Kliegl, Hohenstein, Yan, & McDonald, 2013; Marx, Hawelka, Schuster, & Hutzler, 2015). To illustrate, Hutzler et al. (2013) reported (electrophysiological) evidence that parafoveal X-masks interfere with the processing of a target word. The study by Kliegl et al. (2013) revealed that letter masks induce interference, and concluded that the preview benefit actually is a complex combination of costs and benefits. Marx et al. (2015) corroborated the findings of interference by X-masks and letter masks in samples of young readers. By explicitly referencing to the application of parafoveal masks, Warren, Reichle, and Patson (2011) expressed doubts about the foveal load effect, speculating that Henderson and Ferreira’s “*finding of an interaction may have been related to the interference caused by, and the reprocessing necessitated by, initially processing a nonsense string in the dissimilar preview condition*” (*p*. 8).

**Fig. 1:**
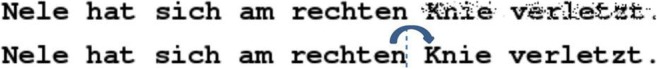
Illustration of the visual degradation of the parafoveal preview – as an alternative to parafoveal masks – in the boundary paradigm. Figure from Marx, Hutzler, Schuster, and Hawelka (2016)

Only a few attempts have been undertaken to investigate the effect of foveal load with alternative means other than the application of parafoveal masks. To our knowledge, Schroyens, Vitu, Brysbaert, and d'Ydewalle (1999) were the first to visually degrade parafoveal previews (rather than applying letter masks) in order to test the foveal load hypothesis (see Fig. 1). Undegraded parafoveal previews elicited a larger preview benefit than degraded previews, confirming that the alternative manipulation affected parafoveal preprocessing as expected. With regard to the foveal load hypothesis, however, Schroyens et al. (1999) replicated the effects reported by Henderson and Ferreira (1990) only partially. They observed a significant interaction of FOVEAL LOAD by PREVIEW for gaze duration, but not for first and single fixation duration. Of note, the task in Schroyens et al. was not a sentence reading task (as in Henderson & Ferreira, 1990), but participants were presented with lists comprising three words and had to indicate whether one of the words was an item of clothing. This difference, too, could have led to the differences in the findings.

A recent study by Marx, Hawelka, Schuster, and Hutzler (2017) also applied visual degradation of parafoveal previews (but in a “standard” sentence reading task) in order to assess whether young readers (children in the 4th and 6th grades) exhibit an effect of foveal load. Similar to the adult participants in the study by Schroyens et al. (1999), the children exhibited shorter first fixation durations and gaze durations on the target words after an undegraded preview than after a degraded preview, indicating that the young readers engaged in parafoveal preprocessing. However, the magnitude of the preview benefit did not differ with regard to the foveal load induced by the pretarget word (i.e., either a high-frequent or a low-frequent adjective). Put differently, Marx et al. (2017) did not observe the LOAD by PREVIEW interaction, which is the critical aspect for the foveal load hypothesis.

### The present study

We can think of three possible reasons why Marx et al. (2017) did not observe the effect of foveal load as predicted by the foveal load hypothesis:

1. Henderson and Ferreira (1990) applied letter masks to manipulate the parafoveal previews. In contrast, Marx et al. (2017) – similar to Schroyens et al. (1999) – visually degraded the previews. The different results could be a consequence of this methodological difference. Indeed, a recent study by Vasilev, Slattery, Kirkby, and Angele (2018) used visually degraded previews in the context of the boundary paradigm with adult readers and did not observe a foveal load effect.
2. The participants in Marx et al. (2017) were children. It might be that young readers do not (yet) show an effect of foveal load, whereas adult readers do. (Note that this would be inconsistent with the above-referenced finding by Vasilev et al., 2018.)
3. The foveal load effect is not replicable. Indeed, direct replications of the effect are scarce. A recent meta-analysis by Veldre and Andrews (2018) reported that only six out of 16 studies, which manipulated the load of a pretarget word (via word frequency) and the preview of the target word, revealed the critical LOAD by PREVIEW interaction. Furthermore, the mean effect size of the interaction in the reviewed studies was small (Cohen’s *d* = 0.09). However, in the empirical part of their study, Veldre and Andrews found the critical LOAD by PREVIEW interaction when they applied orthographically illegal non-words as parafoveal previews (corresponding to the manipulation of Henderson & Ferreira, 1990, and to the one in Exp. 1 in the present study). They did not observe such an interaction with several other kinds of preview manipulations (e.g., real words unrelated to the target words).

The present study examined these potential sources of the discrepancies between the original study by Henderson and Ferreira (1990) and the studies by Marx et al. (2017) and Schroyens et al. (1999). To this end, we applied both the traditional parafoveal masking (as in Henderson and Ferreira; Exp. 1) and the alternative method of visual degradation (as in Schroyens et al. and Marx et al.; Exp. 2) in samples of proficient adult readers. Two levels of foveal load were induced as in the previous studies, that is, by pretarget words of either high or low frequency.

## Method

### Participants

Sixty participants took part in each of the two experiments (48 and 43 female in Exps. 1 and 2, respectively). The participants were primarily University students with a mean age of 23 years (*SD* = 3). They had normal or corrected-to-normal vision; most of them were right-handed (56 out of 60 in both samples), and no one reported a history of a developmental reading deficit or any neurological disorder. The reading speed of the participants was assessed with an appropriate test (see below).

### Material

**Fig. 2:**
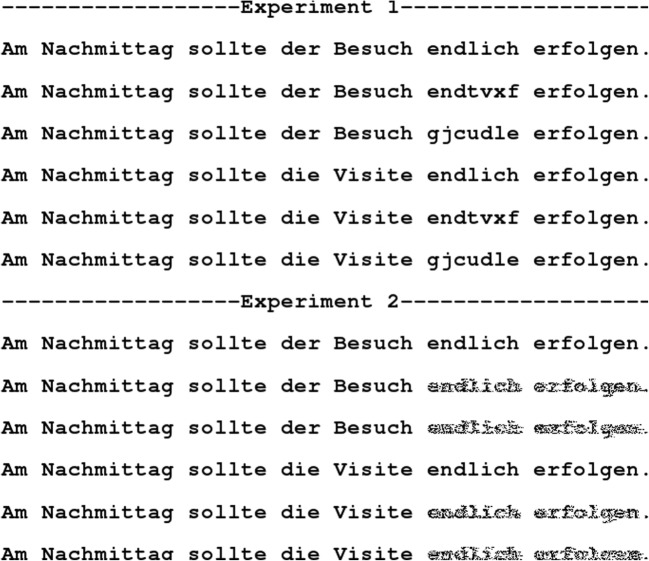
Illustration of the stimuli in Experiments 1 and 2. In the *low foveal load condition* the pretarget word is “Besuch.” In the *high foveal load condition* the pretarget word is “Visite.” (Both words translate to *visit*, but – in German – “Besuch” is much more frequent than “Visite,” with 169 and two occurrences per million, respectively). The invisible boundary was located at the end of the pretarget word. The target word is “endlich” [at last]. The preview of the target was either valid or masked (with a visually similar or dissimilar mask; Exp. 1) or visually degraded (displacement of 33% or 66% of the black pixels; Exp. 2)

In order to realize an experimental design as used by Henderson and Ferreira (1990), we needed an orthogonal combination of the factors frequency of the pretarget word (high vs. low) to manipulate foveal load and type of preview (valid, visually similar and visually dissimilar in Exp. 1 and three levels of visual degradation in Exp. 2) to estimate the preview benefit – resulting in six experimental categories (in each of the two Experiments). For each of the six categories we selected 24 word pairs (i.e., a total of 144 word pairs), each consisting of a low-frequent (LF) and a high-frequent (HF) word. These words were the pretarget words (word_n-1_). For each of these pretarget word pairs we created a sentence frame in which both words fitted contextually. Thus, every sentence exists in two versions concerning the frequency of the pretarget word (word_n-1_). Half of the participants in Experiments 1 and 2 saw the one version of the sentence (e.g., with the HF pretarget word), the other half the complementary one (i.e., with the LF pretarget word). Furthermore, we counterbalanced the 6 × 24 sentence frames across the six experimental categories according to a Latin-square design (each administered to ten participants in each of the Experiments). An example of the sentences is provided in Fig. 2.

The pretarget words (word_n-1_) were adjectives, nouns, or verbs. The frequency of the words was obtained from the *CELEX* database for German (Baayen, Piepenbrock, & Van Rijn, 1993). The criterion for low-frequency was a *log*-frequency of less than 1 (i.e., less than 10 occurrences per million). The criterion for high-frequency was a *log*-frequency greater than 1.4 (> 26 occurrences per million). The means of the *log*-frequency of the two groups were *M* = 0.32 and 1.97, respectively (*SD* = 0.31 and 0.36; t-test group comparison: *t*(286) = 41.73; *p* < .001). The LF and the HF pretarget words were matched on word length (in terms of number of letters; *M* = 6.28 and 6.22 for LF and HF, respectively; *SD* = 1.53 and 1.46; *t*-test group comparison: *t* < 1). The target words (word_n_) were a minimum of five letters long (*M* = 8.14, *SD* = 2.22) and had a mean *log*-frequency of 1.75 (*SD* = 0.80).

The manipulation of the preview of the target word in Experiments 1 and 2 is also illustrated in Fig. 2. As evident from the figure, in Experiment 1 we presented parafoveal masks, replicating Henderson and Ferreira (1990). The three levels of the manipulation of the preview of the target word were *same*, *similar,* and *dissimilar*. In the “same” condition, the preview was identical to the target word. In the “similar” condition, the first three letters were the same as in the target word and the other letters were visually similar (i.e., letters with ascenders were replaced by another ascender; descenders with descenders). In the “dissimilar” condition, the previews were visually dissimilar letter strings (e.g., ascenders replaced by descenders). In Experiment 2, the previews were visually degraded – similar to Marx et al. (2017). The visual degradation ranged from 0% degradation (no pixel replacement) via a medium degradation (33% of the black pixels were displaced) to a high degradation (66% pixel displacement). The degradation was implemented with the help of the *pixmap-package* (Bivand, Leisch, & Mächler, 2011) and an *R-script*. Note that not only the letters of the target word were degraded, but also the remainder of the sentence (similar to Marx et al., 2017).

We created the sentences in such a way that a minimum of four words preceded the pretarget word; a minimum of one word succeeded the target word. All sentences were 7–15 words long (*M* = 9.6) and were presented in a bold and mono-spaced font type in black on a white background. In terms of number of characters (including blank spaces), the sentence had a mean length of 62 characters (*SD* = 9). A single character had a width of ~0.4° of visual angle. The participants were familiarized with the experiment with 12 practice trials. Twelve sentences (i.e., ~ 8%) of the experiment were followed by questions (verbally provided by the experimenter) to maintain the vigilance of the participants and to ensure that they adhered to the instruction to read the sentences for comprehension.

#### Target word predictability

The sentence frames – in which the pretarget word (which was manipulated with regard to its frequency) and the target word were embedded – were identical for the two levels of foveal load. However, it still could be that a low-frequency pretarget word induces a different predictability of the target word than a high-frequency pretarget word. To assess this possibility, we conducted a sentence completion task (*cloze task*; Taylor, 1953) in independent norming samples (*n* = 24 and 23 for the low-load and the high-load version of the sentences, respectively). In this task, the participants were presented with the words of the sentences up to the pretarget word and had to predict the continuation (i.e., the target word). In the low-load and the high-load versions of the sentences the target word was predicted correctly with *p* = .17 and .15, respectively. An independent sample t-test revealed that the difference in predictability was not significant; *t*(286) < 1.

#### Assessment of reading performance

A potential reading disorder was defined as exhibiting a reading rate worse than the fifth percentile of the population of students and was assessed with an (unpublished) student version of the *Salzburger Lesescreening* (SLS; Wimmer & Mayringer, 2014). The test requires the subject to silently read sentences and to judge them as meaningful (e.g., “A week has seven days”) or incorrect (e.g., “A weighing machine measures the height of a person”) within a time limit of 3 min. The incorrect statements were obvious violations of common knowledge, so that judging the correctness was easy (*M* < 1 incorrect marking in both groups). Therefore, the measure (i.e., the number of correctly marked sentences) is an index of reading speed. The preliminary norms of the test are based on a sample of 309 university students. Compared to the norm, the average performance of the present participants corresponds to percentile 64 (for the participants in both Exp. 1 and Exp. 2). The reading performance of the slowest readers (one in each of the two samples) corresponds to percentile 9.

### Apparatus

We recorded the eye movements for the right eye with an EyeLink 1000 (SR Research, Ottawa, Ontario, Canada) with a sampling rate of 1,000 Hz. The stimuli were shown on a screen with a resolution of 1,366 × 768 and a 144-Hz frame rate. The viewing distance of 52 cm was held constant by a forehead and a chin rest.

### Procedure

The participants were randomly assigned to either Experiment 1 or Experiment 2 (*n* = 60 in each Experiment). The eye tracker was calibrated with a horizontal 3-point calibration routine. The calibration was conducted before the familiarization trials and was repeated before the main experiment and after every comprehension question during the experiment (i.e., at least 12 times during the experiment). The criterion for a successful calibration was an average tracking error below 0.3° of the visual angle. A fixation cross (12 × 12 pixels – centered on the first word of the upcoming sentence) preceded the presentation of each sentence. When the eye-tracking system detected (within a time-limit of 5 s) a fixation on the cross, the sentence was presented; otherwise the eye tracker was re-calibrated. The participants were instructed to silently read the sentences for comprehension and to terminate each trial by button press, whereupon the next fixation cross or a question mark appeared. The comprehension questions mostly asked for nouns (subjects or objects) and twice for an adverb. For example, the sentence “*In order not to wake anyone, they moved slowly through the house*” was followed by the question “*How did they move through the house?*”. The questions were verbally provided by the experimenter and the participant had to reply verbally, too. Most questions could be answered by uttering a single word. After the participant had answered, the examiner logged the correctness of the answer and the eye tracker was re-calibrated. After the experiment, the participants were asked if they had noticed “something uncommon” concerning the presentation of the sentences to check if they had noticed the display change.

### Data treatment and analyses

We analyzed first fixation duration (FFD), single fixation duration (SFD), and gaze duration (GD) on the pretarget and the target word. The pretarget word analysis served as a manipulation check and revealed whether our manipulation of foveal load by word frequency elicited the expected effect, that is, significantly longer fixation times on low-frequent than on high-frequent pretarget words. Additionally, the analysis revealed potential parafoveal-on-foveal effects, that is, effects of the type of preview on fixation times on the pretarget word. The theoretically relevant aspect is the analysis of the inspection times on the target words. Finding an interaction of FOVEAL LOAD (induced by the pretarget word) and TYPE OF PREVIEW (of the target word) would be indicative for the foveal load effect. For the analyses, we *log*-transformed the fixation time measures, but the figures display them in the original metric.

We analyzed the data by means of linear mixed models (LMM) using the *lme4* package (version 1.1-12; Bates, Maechler, Bolker, & Walker, 2015) in the *R* environment for statistical computing. In general, the models included LOAD and TYPE OF PREVIEW as fixed effects and subjects and items as random effects. We estimated random effects on the intercepts and for the slopes of the experimental contrasts (reliable variance components associated with intercepts and contrasts were included; correlation parameters specified as zero). As the first contrast (C1), we compared the valid preview condition with the average of the two invalid preview conditions. The second contrast (C2) is the comparison of the two invalid conditions, that is, the “subtle” violation of the preview (similar preview and 33% degradation in Exps. 1 and 2, respectively) and the “strong” violation of the preview (dissimilar preview and 66% degradation). These Helmert contrasts yield two tests of the LOAD by PREVIEW interaction. The more critical interaction, however, is the one between the valid preview and the average of the invalid/degraded previews (i.e., contrast C1). t-Values greater than 1.96 were considered significant.

We considered fixation durations shorter than 80 ms or longer than 600 ms as outliers. For gaze duration, we chose a cut-off of 1,250 ms. Only trials in which both the pretarget and the target word received a (valid) fixation and in which the incoming saccade on the target word originated from the pretarget word were considered in the analyses. These criteria resulted in the omission of 17% and 21% of the trials in Experiments 1 and 2, respectively. For the measure of FFD, this left us with 7,072 and 6,743 valid observations in Experiments 1 and 2, respectively. For SFD and GD the figures were 4,002 and 6,989 (Exp. 1) and 4,207 and 6,673 (Exp. 2).

## Results

### Sentence comprehension

The participants answered a minimum of nine (out of 12) comprehension questions correctly with means of 11.4 and 11.2 in Experiments 1 and 2, respectively. This close-to-ceiling performance confirms that the participants adhered to the instruction to read the sentences for comprehension.

### Pretarget word (word_n-1_)

**Fig. 3:**
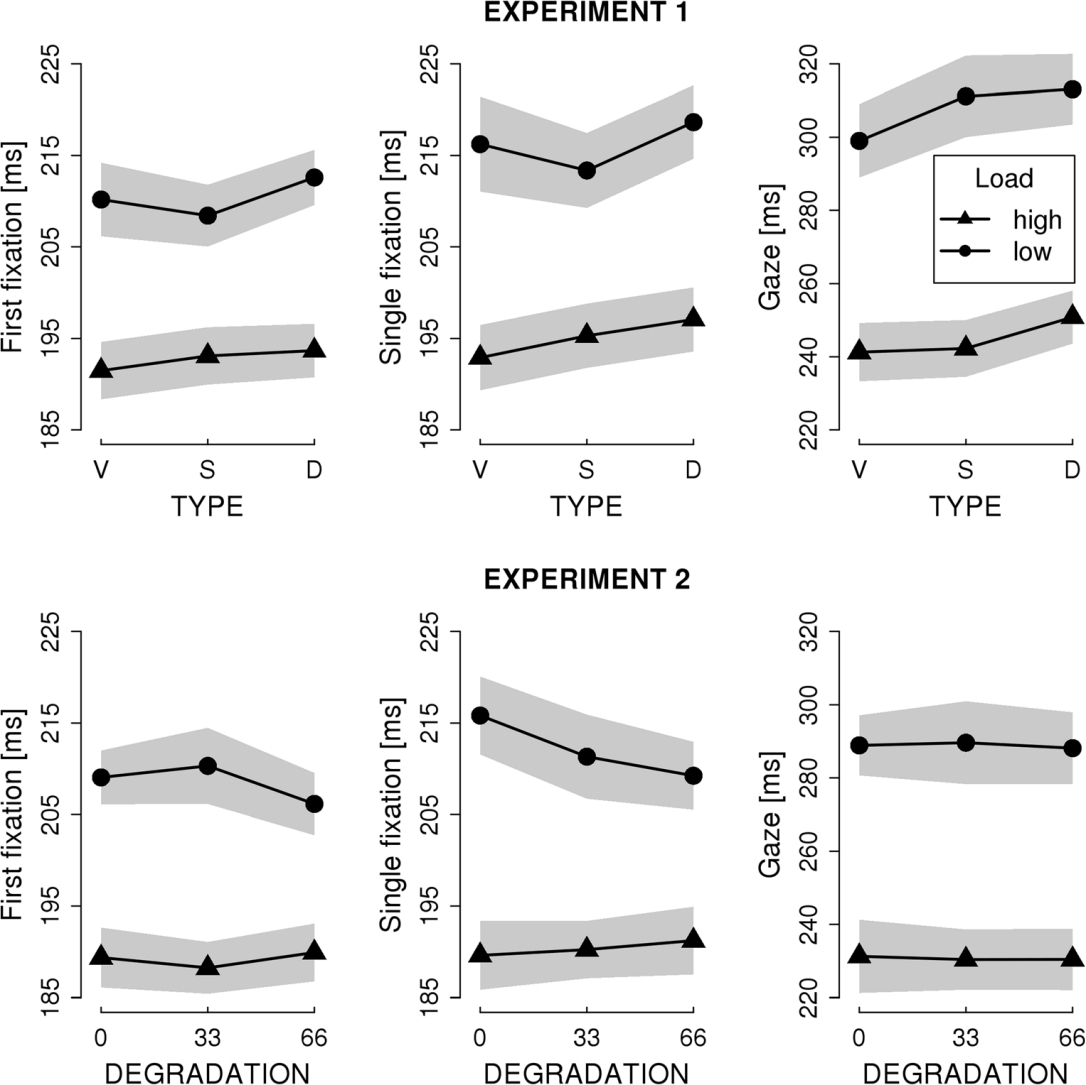
Mean fixation times on the **pretarget word.** The separate lines show the means for low-load (i.e., high frequency) and high-load (low-frequency) pretarget words. The x-axis shows the levels of the type of preview of the target words. The levels are valid (V), similar (S), and dissimilar (D) in Experiment 1 and 0%, 33%, and 66% of visual degradation in Experiment 2. The shaded areas depict the 95% within-subject confidence intervals (cf. Cousineau, 2005)

Fixation times on the pretarget words are illustrated in Fig. 3. The results from the LMM analysis are presented in Table 1. Figure 3 indicates longer fixation times on low-frequency (i.e., high load) than high-frequency (i.e., low load) pretarget words. Indeed, Table 1 reveals that the main effect of LOAD was significant for all measures in both Experiments. The significant frequency effect confirms the induction of the different levels of foveal load. Apart from the effect of LOAD, we observed some significant parafoveal-on-foveal effects for previewing letter-masks in Experiment 1. As evident from the Table and Fig. 3, previewing dissimilar letter masks elicited longer SFD than previewing letter masks that were visually similar to the upcoming target word. Likewise, GD were longer for dissimilar than similar letter masks and similar letter masks elicited longer GD than the valid previews. No such parafoveal-on-foveal effects were observed for visually degraded previews (i.e., in Exp. 2). Neither in Experiment 1 nor in Experiment 2 did we observe significant LOAD by PREVIEW interactions.

**Table 1:** Results of the linear mixed models for fixation measures on the pretarget word. Significant effects are indicated with an asterisk

Fixed effect	Experiment 1			Experiment 2		
	b	SE	t	b	SE	t
First fixation duration						
Intercept	5.262	0.015	360.0*	5.244	0.017	315.4*
Load (i.e., pretarget frequency)	-0.042	0.005	-8.02*	-0.046	0.005	-8.66*
C1: Valid vs. invalid preview	-0.002	0.002	-0.94	0.002	0.002	0.70
C2: Similar vs. dissimilar preview	-0.006	0.004	-1.57	0.003	0.004	0.84
Load X C1	-0.001	0.002	-0.65	-0.001	0.002	-0.36
Load X C2	0.001	0.004	0.25	-0.006	0.004	-1.48
Single fixation duration						
Intercept	5.288	0.015	353.3*	5.26	0.017	302.2*
Load (i.e., pretarget frequency)	-0.052	0.006	-8.55*	-0.054	0.006	-9.10*
C1: Valid vs. invalid preview	-0.003	0.003	-1.02	0.003	0.003	1.00
C2: Similar vs. dissimilar preview	-0.008	0.001	-2.10*	0.002	0.004	0.46
Load X C1	-0.003	0.002	-1.37	-0.005	0.002	-1.95
Load X C2	0.001	0.004	0.24	-0.001	0.004	-0.36
Gaze duration						
Intercept	5.488	0.024	230.9*	5.429	0.024	223.7*
Load (i.e., pretarget frequency)	-0.105	0.011	-9.62*	-0.099	0.011	-9.43*
C1: Valid vs. invalid preview	-0.009	0.004	-2.34*	0.001	0.004	0.26
C2: Similar vs. dissimilar preview	-0.014	0.006	-2.37*	0.001	0.006	0.11
Load X C1	-0.001	0.003	-0.15	-0.002	0.003	-0.58
Load X C2	-0.004	0.006	-0.61	0.001	0.006	0.18

### Target words (word_n_)

**Fig. 4:**
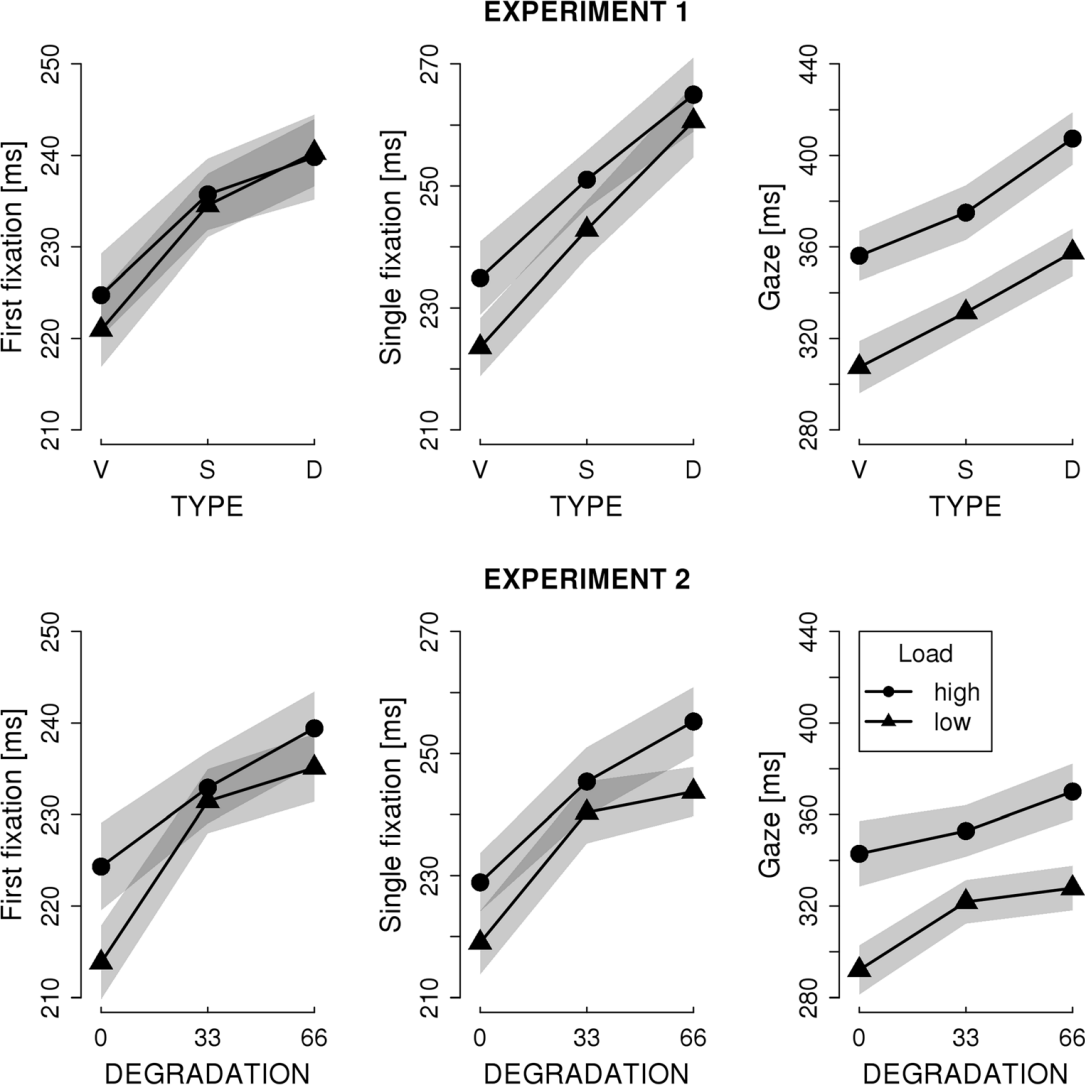
Mean fixation times on the **target word.** The separate lines show the means for low-load (i.e., high frequency) and high-load (low-frequency) pretarget words. The x-axis shows the levels of the type of preview of the target words. The levels are valid (V), similar (S), and dissimilar (D) in Experiment 1 and 0%, 33%. and 66% of visual degradation in Experiment 2. The shaded areas depict the 95% within-subject confidence intervals (cf. Cousineau, 2005)

Fixation times on the target words are illustrated in Fig. 4. The results from the LMM analysis are presented in Table 2. Figure 4 indicates that fixation times were shorter after low-load inducing (i.e., high-frequency) pretarget words than after high-load (low-frequency) pretarget words. Indeed, the main effect of LOAD was significant across experiments and measures except for FFD in Experiment 1. Figure 4 also indicates that the type of preview affected fixation durations. In both Experiments, fixation durations were shortest for the valid preview and increased with increasing deviance of the preview from the actual target word. The respective contrasts presented in Table 2 (C1: valid preview condition vs. the average of the two invalid preview conditions; C2: the visually similar / 33% degraded vs. the visually dissimilar / 66% degraded previews) are all significant. With respect to the critical LOAD by PREVIEW interaction, the evidence is mixed. Rather unexpectedly, we obtained the critical interaction for the visually degraded previews (Exp. 2), but not for the letter masks (Exp. 1). The critical interactions in Exp. 2 were evident for FFD and GD, but not for SFD (but this could be due to the smaller number of observations as instances of single fixations are rarer than instances of the other two measures). Furthermore, only the contrast of the valid preview condition with the average of the two invalid preview conditions (C1) revealed LOAD by PREVIEW interactions. The fixation times after previewing a 33% versus a 66% degraded preview were not significantly modulated by LOAD. Moreover, although the critical contrast (C1) revealed significant LOAD by PREVIEW interactions in Experiment 2, the effect seems to be small (see Fig. 4). The estimation of effect sizes (for repeated measures; Dunlap, Cortina, Vaslow, & Burke, 1996) revealed *d* = 0.14 and 0.17 for SFD and GD, respectively. (For comparison, the effect sizes of the main effect of LOAD for SFD and GD were *d* = 0.26 and 0.53 in Experiment 1 and 0.21 and 0.61 in Experiment 2.)

**Table 2:** Results of the linear mixed models for fixation measures on the target word. Significant effects are indicated with an asterisk

Fixed effect	Experiment 1			Experiment 2		
	b	SE	t	b	SE	t
First fixation duration						
Intercept	5.403	0.017	323.8*	5.39	0.016	354.9*
Load (i.e., pretarget frequency)	-0.002	0.004	-0.50	-0.009	0.004	-2.19*
C1: Valid vs. invalid preview	-0.218	0.003	-6.63*	-0.026	0.004	-6.79*
C2: Similar vs. dissimilar preview	-0.008	0.004	-2.16*	-0.011	0.004	-2.89*
Load X C1	-0.003	0.002	-1.39	-0.005	0.002	-2.16*
Load X C2	-0.003	0.004	-0.65	0.000	0.004	0.02
Single fixation duration						
Intercept	5.482	0.018	298.2*	5.45	0.018	300.3*
Load (i.e., pretarget frequency)	-0.019	0.005	-3.76*	-0.018	0.005	-3.41*
C1: Valid vs. invalid preview	-0.037	0.004	-9.01*	-0.034	0.004	-7.63*
C2: Similar vs. dissimilar preview	-0.032	0.004	-7.15*	-0.014	0.004	-3.54*
Load X C1	-0.004	0.003	-1.68	-0.004	0.002	-1.62
Load X C2	-0.005	0.004	-1.15	0.003	0.004	0.81
Gaze duration						
Intercept	5.748	0.027	210.0*	5.69	0.026	221.3*
Load (i.e., pretarget frequency)	-0.061	0.008	-7.22*	-0.056	0.009	-6.40*
C1: Valid vs. invalid preview	-0.042	0.005	-8.65*	-0.032	0.006	-5.46*
C2: Similar vs. dissimilar preview	-0.044	0.006	-7.56*	-0.032	0.006	-2.99*
Load X C1	-0.006	0.003	-1.66	-0.009	0.003	-2.59*
Load X C2	0.001	0.006	0.15	0.004	0.006	0.76

The most critical measure for the foveal load hypothesis is the interaction of LOAD with the TYPE OF PREVIEW (i.e., the valid preview condition versus the average of the two invalid preview conditions) which is denoted as Load X C1

### Display change awareness

As yet, we analyzed the data regardless of the participants’ awareness of the display changes in the boundary paradigm. Henderson and Ferreira (1990) only considered trials in which the participants could not perceive the display change. White, Rayner, and Liversedge (2005) observed evidence indicative of a foveal load effect only in the group of participants who did not detect the display changes. We also asked our participants whether they noticed “something uncommon.” In Experiment 1, 34 of the 60 participants were aware of display changes. In Experiment 2, the vast majority of the participants (46) noticed the display changes, that is, only 14 participants were unaware of the changes.

We entered display change awareness as an additional fixed effect into the LMM analysis for the target words. For Experiment 1, the main effect of display change awareness was not significant for any of the measures (i.e., SFD, FFD, and GD); all |*t*|s ≤ 1.42. The awareness of the display changes, however, interacted with TYPE OF PREVIEW for the measures SFD and GD; *t* = 3.39 and 2.44, respectively (for FFD, this interaction was not significant; *t* = 1.9). The interaction was such that the participants, who were aware of the display changes, exhibited much longer SFD and GD in the dissimilar preview condition (*M* = 268 and 401 ms, respectively) than in the similar preview condition (*M* = 243 and 361 ms) compared to the participants who were not aware of the display changes (257 and 361 ms for dissimilar previews vs. 251 and 344 ms for similar previews). Put differently, the “aware participants” were more “sensitive” to (or more “disrupted” by) the dissimilar previews than the “unaware participants.” Critically, however, the triple interactions DISPLAY CHANGE AWARENESS by LOAD by TYPE OF PREVIEW were all insignificant; |*t*|s ≤ 1.6. For Experiment 2 and for all measures, display change awareness had no significant effect whatsoever, neither as a main effect nor in interaction with the other factors (all |*t*|s ≤ 1.6).

## Discussion

In two Experiments, we assessed the foveal load hypothesis, namely, whether the processing difficulty of the currently fixated word modulates the extent of parafoveal preprocessing of the upcoming word (Henderson & Ferreira, 1990). The present study was motivated by the finding of Marx et al. (2017), who (amongst others; see below) could not replicate the effect of foveal load. One possible explanation could be that Marx and colleagues applied a non-standard experimental manipulation of the parafoveal preview of the target words. They visually degraded the previews (similar to Schroyens et al., 1999), whereas the original study on the foveal load effect applied parafoveal letter masks. Another possible explanation is that Marx et al. (2017) assessed the effect of foveal load in children, whereas the original study assessed the effect in adult readers.

In the present study we investigated these potential explanations by applying both parafoveal letter masks (i.e., the standard experimental manipulation of the parafoveal previews; Exp. 1) and visually degraded parafoveal previews (i.e., the alternative manipulation; Exp. 2). Furthermore, we assessed the effect in adult readers. In Experiment 1, we tried to replicate the original study as directly as possible: (1) the parafoveal masks were created in the same way as in Henderson and Ferreira; (2) the same principle was applied for selecting high-load and low-load inducing words, which differed in frequency, but were pairwise fitted into the same sentence frames (mostly by being synonyms). Using word frequency in order to experimentally manipulate foveal load is the standard manipulation not only in Henderson and Ferreira but also in, for example, Drieghe, Fitzsimmons, and Liversedge (2017), Schroyens et al. (1999), and White et al. (2005).

The findings of the present study with regard to the critical LOAD by PREVIEW interaction are mixed. Rather unexpectedly, we found some evidence for the effect of foveal load in Experiment 2 in which we applied the alternative manipulation of the parafoveal preview, that is, visual degradation. Previous studies using a similar manipulation reported no (Marx et al., 2017) or only ambiguous (Schroyens et al., 1999) evidence for the effect of foveal load. A first implication of the present finding in adult readers is that young readers – to whom Marx et al. administered a similar experiment – may indeed not (yet) exhibit an adult-like effect of foveal load on the amount of parafoveal preprocessing. This is an issue that warrants further investigation.

In Experiment 1, in which we applied the standard manipulation of the previews, that is (visually similar and dissimilar) letter masks, we did not find a significant effect of foveal load. This failure to replicate the original study by Henderson and Ferreira (1990) is in line with several other studies that could not (conceptually) replicate the effect: Drieghe, Rayner, and Pollatsek (2005) and Drieghe et al. (2017), for example, did not find a LOAD by PREVIEW interaction on fixation times. White (2007) as well as Drieghe et al. (2005) did not find a LOAD by PREVIEW interaction for word skipping. Veldre and Andrews (2018), who systematically reviewed the literature on the foveal load effect, reported that only six of 16 pertinent studies found the critical LOAD by PREVIEW interaction.

One may speculate that the fickle nature of the effect of foveal load on parafoveal preprocessing relates to the application of parafoveal letter masks. These masks could be a suboptimal means to manipulate the availability of parafoveal information. As mentioned in the Introduction, such a reservation was expressed by Warren et al. (2011; see also Luke, 2018; Veldre & Andrews, 2018), and recent evidence indeed suggests that parafoveal letter masks may induce uncalled-for effects (Kliegl et al., 2013; Marx et al., 2015). The analyses in the present study on the pretarget word revealed that letter masks elicit parafoveal-on-foveal effects that may, as a consequence, alter the processing of the target word once it is fixated. These parafoveal-on-foveal effects were a prolonged SFD on the pretarget word when the preview was a dissimilar letter mask and a longer GD for both similar and dissimilar letter masks than for valid previews. For previewing visually degraded target words, we did not observe similar effects. Whether, and, if so, how such parafoveal-on-foveal effects of letter masks affect parafoveal preprocessing, in general, and the effect of foveal load, in particular, require further investigation.

Another possibility for the fickleness of the foveal load effect could be that the load induced by word frequency is simply too weak. In principle, our experimental manipulation worked – at least in the expected direction (if not to a sufficient amount): The analyses on the pretarget word revealed substantially longer fixation times for low-frequency than for high-frequency words. However, it may still be that a word frequency manipulation is insufficient (or even ill-suited) to elicit those differences in foveal load that would be required to (consistently) produce the effect. Veldre and Andrews (2018) reported that the mean effect size of the LOAD by PREVIEW interaction – in studies that induced the load with a word frequency manipulation – is only *d* = 0.9. The maximum effect size (the one from the original Henderson & Ferreira study) was *d* = 0.14. This figure fits well with the effects sizes that we obtained in Experiment 2. The diminutiveness of the foveal load effect is certainly a reason for the repeated failures to replicate it.

The attentional span in reading, however, may well be dynamic and modulated by load, but word frequency is simply not a sufficiently powerful variable to (consistently) induce significant differences in foveal load. Henderson and Ferreira (1990) already demonstrated that the induction of different levels of foveal load by means of syntactic difficulty elicits differences in parafoveal preprocessing. Payne, Stites, and Federmeier (2016) showed that foveal semantic load modulates parafoveal preprocessing. Kaakinen and Hyönä (2014) showed that different task instructions can induce different levels of load. It is also plausible that the dynamic modulation of the attentional span in reading does not necessarily translate to differences in fixation duration, but can be captured by other measures (e.g., Ghahghaei & Linnell, 2018). Furthermore, the effect is probably more apparent in non-alphabetic orthographies (e.g., Yan, Kliegl, Shu, Pan, & Zhou, 2010). Finally, the size of the effect may differ in participants with different levels of reading proficiency (Veldre & Andrews, 2015).

Several factors have been suggested that might modulate the critical LOAD by PREVIEW interaction. Participants’ awareness for display changes, for example, was invoked as a restricting factor. Henderson and Ferreira (1990) only analyzed trials in which the participants were unaware of the display change. White et al. (2005) observed an effect of foveal load only in the subgroup of participants who were not aware of the display change. In the present study, however, the LOAD by PREVIEW interaction was not modulated by the display change awareness of the participants – a finding paralleling the recent evidence by Veldre and Andrews (2018).

It is of note that the visual degradation of the parafoveal preview elicited much higher display change awareness than the orthographic manipulation in Experiment 1. On the one hand, this is surprising as the study by Marx et al. (2017) on the foveal load effect in children, which used a similar manipulation, reported a very low rate of participants who were aware of the display changes. On the other hand, this finding is in agreement with the study by Vasilev et al. (2018), in which a very high awareness for visually degraded previews was reported . Interestingly, Vasilev and colleagues did not find an effect of foveal load when applying visual degradation as the manipulation of the parafoveal preview. They interpreted the absence of the effect as an indication that visual degradation is a suboptimal manipulation for studying parafoveal preprocessing in reading. In the light of the present study and the review of Veldre and Andrews, we may remark that judging the suitability of an experimental manipulation on the basis of a fickle effect is (admittedly, with the benefit of hindsight) questionable.

Besides the high awareness of the display changes, there is another commonality between the study by Vasilev et al. (2018) and the present one. Similar to our study, Vasilev and colleagues reported that visually degraded previews did not induce parafoveal-on-foveal effects (see above), whereas parafoveal letter masks are prone to elicit such effects as demonstrated by the present study (see also Hutzler et al., in press). Put differently, traditional letter masks generate more preview cost than visual degradation. Thus, if the awareness of the display changes could be reduced (an anonymous reviewer suggested diminishing the stimulus-background contrast), then visual degradation may indeed be a better manipulation of parafoveal previews than the traditional letter masks.

Whereas the evidence for a foveal load effect (within the meaning of Henderson & Ferreira, 1990) is mixed in the present study, another effect turned out to be reliable. The mean SFD and GD (and, in Exp. 2, also FFD) on the target words were significantly longer after low-frequent than after high-frequent pretarget words (i.e., we found a main effect of LOAD). Put differently, we observed a *spillover* effect (also known as *lag effect*; e.g., Kliegl, Nuthmann, & Engbert, 2006). In the literature, the foveal load and the spillover effect are often not sufficiently distinguished conceptually, but are treated as the two sides of the same coin. These effects, however, are conceptually different. The foveal load effect relates to parafoveal preprocessing of the upcoming word. The spillover effect, in contrast, relates to ongoing processing of the previous word. To be specific, the effect is considered to arise when a reader has not yet fully processed a word but he/she already fixates the next word. In other words, processing of one word *spills over* to the next word – a process implemented in the SWIFT model of eye-movement control during reading (Engbert, Nuthmann, Richter, & Kliegl, 2005).

The SWIFT model assumes that the generation of saccades is autonomous during reading, but saccades can be inhibited when a reader encounters difficulties during word recognition (e.g., when he/she encounters a low-frequency word). However, (cortical) processing of words is assumed to be slower than the autonomous generation of saccades (in the brain stem). Thus, the inhibition of saccades – which results in a prolongation of fixations – may occur in a time-delayed manner, that is, when the reader is already ahead of the word that induced the inhibition. Although it is beyond the scope of the present study, we believe that this explanation of the spillover effect is more parsimonious than the explanation provided by the E-Z Reader model (Reichle, Pollatsek, Fisher, & Rayner, 1998). The latter model explains the spillover effect in accordance with the foveal load hypothesis, that is, by assuming reduced parafoveal preprocessing during the fixation on the preceding word, which, in turn, is induced by the difficulty of recognizing this word (e.g., Reichle, Rayner, & Pollatsek, 2003).

To sum up, the present study found mixed evidence for the foveal load effect. We could not observe the effect when we applied parafoveal letter masks as the manipulation of the previews, which is the traditional and standard approach to investigate the effect. In contrast, we did observe the effect when we applied visual degradation of the previews. This method, hitherto, has not produced much affirmative evidence for the foveal load hypothesis (Marx et al., 2017; Schroyens et al., 1999). These findings (together with those of Veldre & Andrews, 2018 and Vasilev et al., 2018) demonstrate that the baseline (i.e., the experimental manipulation of previews) is an important issue when studying the effect of foveal load. Furthermore, while word frequency is the most common, it is certainly not the strongest manipulation possible for inducing different levels of foveal load. The essential conclusion of the present study, thus, is that the frequency-based foveal load effect is quite small, and hence its reproducibility is uncertain (Drieghe et al., 2017). Future investigation may consider alternative baseline contrasts (i.e., alternatives to parafoveal letter masks) and alternative means to induce foveal load.
